# The lasting effects of fear of childbirth: parous women’s experiences of postponing or avoiding subsequent pregnancies

**DOI:** 10.1186/s12978-025-02261-1

**Published:** 2026-01-09

**Authors:** Elin Ternström, Nora Aarseth, Elisabet Rondung

**Affiliations:** 1 School of Health and Welfare, Dalarna University, Falun, 791 88 Sweden; 2 Department of Women’s and Children’s Health, Uppsala University, Uppsala, Sweden; 3 Department of Psychology and Social work, Mid Sweden University, Östersund, Sweden

**Keywords:** Birth, Fear of childbirth, Maternity care, Pregnancy, Traumatic birth experience, Women’s experiences

## Abstract

**Background:**

In research and clinical practice, fear of childbirth (FOC) is mainly identified and acknowledged during pregnancy. Hence, knowledge about how FOC affects women before and between pregnancies is limited. Here, we aimed to explore the experience of FOC among non-pregnant women who had previously given birth but were hesitant to become pregnant again despite a strong desire for more children.

**Methods:**

This study was part of a larger qualitative interview study exploring FOC among non-pregnant women and builds on eight semi-structured interviews. All participating women had given birth once or twice before. They longed for one or more children but were hesitant about another pregnancy and birth due to FOC. Six of them had experienced FOC during their last pregnancy. We analyzed the interviews using reflective thematic analysis.

**Results:**

Through analysis, we identified five themes: “Fear disturbs my daily life and makes me avoid another pregnancy,” “Negative perinatal care experiences fueled my pre-existing fears,” “I want to control the mode of birth as I expect a worst-case scenario,” “I need professional support even if I’m not pregnant,” and “See me for who I am and listen to my specific needs.” These were summarized under the overarching theme: “In the grip of fear, striving to find a way out.” Despite the women’s desire for more children, fear led them to delay or avoid future pregnancies. This fear often originated before or during their first pregnancies and was sometimes intensified by traumatic childbirth experiences. A lack of professional support and individualized care left them feeling isolated and unsupported, which significantly impacted their lives and reproductive futures, illustrating their ongoing struggle to escape the grip of fear.

**Conclusions:**

The findings highlight an urgent need for support systems, addressing the psychological needs of women with FOC, not only during pregnancy but whenever support is needed throughout their reproductive lives. To help women manage their FOC and regain confidence in giving birth, it is essential to respect their individual concerns, choices, and preferences. Maternal care providers must be equipped to offer a broader range of options within maternal care to meet these needs.

**Supplementary Information:**

The online version contains supplementary material available at 10.1186/s12978-025-02261-1.

## Abstract (plain language summary)

Fear of childbirth (FOC) is usually recognized during pregnancy, but we don’t know much about how it affects women before and between pregnancies. This study explored the experiences of non-pregnant women who had previously given birth but were hesitant to become pregnant again due to FOC, even though they wanted more children. The study involved eight interviews with women who had given birth once or twice. These women wanted more children but were uncertain about facing another pregnancy because of FOC. Six of them had experienced FOC during their last pregnancy.

The analysis showed that the women’s fear often started before or during their first pregnancies and was sometimes worsened by traumatic birth experiences. The women also emphasized the need for professional support even when not pregnant and stressed the importance of being seen, heard, and treated as individuals. These experiences had a big impact on their lives and future pregnancies, showing their ongoing struggle to overcome their fear.

The study highlights that women with FOC need psychological support throughout their reproductive period, not just during pregnancy. To help women manage their FOC and feel more confident about childbirth, it’s important to respect their unique concerns, choices, and preferences. Maternal care providers should be prepared to offer various options to meet these needs.

**Supplementary Information:**

The online version contains supplementary material available at 10.1186/s12978-025-02261-1.

## Background

Globally, fear of childbirth (FOC) affects many women negatively throughout their reproductive lives [1]. There is no clear, universally accepted definition of FOC. Although early research defined it as fear occurring during pregnancy and linked to the upcoming birth [2], later studies have demonstrated that FOC can arise even before pregnancy, as early as adolescence [3, 4].

Although research on FOC outside of pregnancy is sparse, studies among non-pregnant female university students from Australia, the United States, and Croatia have reported a prevalence of 26–27% [5–7]. When examining the nature of their fears more closely, fear of bodily harm, intense pain, complications, and loss of control during childbirth are concerns frequently mentioned [8]. These fears are also commonly reported by pregnant women experiencing FOC [9]. Our previous research has shown that women who wanted to start a family but were afraid to become pregnant due to FOC expressed similar fears. They also reported having limited access to support, which left them alone to deal with their fears and unable to decide whether to become pregnant [4]. While these studies address FOC before a first pregnancy, we are not aware of any studies exploring FOC among women who have previously given birth but experience intense fear at the thought of giving birth again.

Among women who have given birth, FOC is often linked to a previous traumatic or operative birth [10] and is commonly labeled secondary FOC. Several individual vulnerability factors, along with specific events during childbirth, may interact to determine the appraisal of birth as traumatic [11, 12]. In retrospect, women with negative or traumatic birth experiences often describe feeling detached or absent, powerless, out of control, and incapable of giving birth. Negative interpersonal experiences during childbirth are also commonly reported, e.g., being denied control, lacking a voice, feeling excluded, dismissed, dehumanized, and not receiving sufficient support [13–15]. A traumatic childbirth experience could thus be defined as “a woman’s experience of interactions and/or events directly related to childbirth that caused overwhelming distressing emotions and reactions; leading to short and/ or long-term negative impacts on a woman’s health and wellbeing” [16, p.691].

Despite the importance of previous birth experiences, we are aware of only one longitudinal study that measured FOC over two pregnancies. That study disclosed that, after adjusting for sociodemographic variables and other risk factors, FOC during the first pregnancy was the strongest predictor of FOC in a subsequent pregnancy [17]. This suggests that some women with secondary FOC may have also experienced primary FOC that went unrecognized, untreated, or unresolved during their first pregnancy and birth. There is also evidence showing a clear association between FOC during pregnancy and a more negative birth experience [18, 19], indicating the possibility of a vicious cycle of fear.

Among women with FOC who have given birth more than once, studies have shown that they tend to have longer intervals between births compared to non-fearful control groups [20, 21]. Additionally, negative birth experiences have also been associated with postponed pregnancies, fewer subsequent births, and a reduced number of children overall [22, 23]. This suggests that these women likely experienced FOC between pregnancies, making them more inclined to avoid or postpone future pregnancies and childbirths. Such postponement has been documented previously [24, 25] and was briefly described by Hofberg and Brockington [3] (p.84): “The dilemma for these women was that the family felt incomplete but the women were terrified of a further delivery.”

In conclusion, research on FOC has mainly focused on pregnant women, leaving a gap in our understanding of how FOC affects women before and between pregnancies. In this study, we focus on cases where FOC hinders the pursuit of additional pregnancies among women who have previously given birth. Our aim was to explore the experiences of non-pregnant women who, despite a strong wish to have more children, feel hesitant to become pregnant again. These women may or may not have had previous traumatic birth experiences.

## Methods

This qualitative study is part of a larger research project exploring FOC among non-pregnant women. A recently published article from the same project focused on women without prior birth experience [4], whereas the present study addresses women with previous birth experience. Data were collected through semi-structured interviews with eight non-pregnant women with FOC. All had previously given birth and expressed a longing for one or more additional children, but felt hesitant about another pregnancy and birth due to FOC. The first author is a licensed midwife, and the last author is a licensed psychologist; both are active researchers within the fields of FOC and traumatic birth experiences. The second author, who conducted the interviews, was a master’s student in clinical psychology at the time of the study. None of the researchers had any previous relationships with the participants.

### Setting

In Sweden, national guidelines recommend screening for FOC several times during pregnancy, for example, by using the Fear of Birth Scale [26]. If FOC is identified during pregnancy, most hospitals offer midwife-led counseling through a specialized FOC unit. This counseling is free of charge and is usually provided by specially trained midwives and obstetricians; however, social workers, psychologists, and psychotherapists may also be part of the care team [27] or offer support and treatment in other settings. Women in Sweden do not have the freedom to choose their mode of birth. If a pregnant woman requests a cesarean section, she is referred to a FOC unit, where an obstetrician determines the appropriate mode of birth [28]. After birth, some FOC units provide follow-up care for women who have had negative birth experiences, but the organization and provision of this care differ across regions [29].

### Recruitment

As part of the larger research project, participants were recruited through posts published on Instagram and Facebook between February 18 and 24, 2020. Those interested could click on a link in the post, which directed them to a web-based registration form. The form commenced with information about the study, after which prospective participants could provide their consent to participate and submit background and contact information. Background information, including age, marital status, country of birth, size of hometown, and educational level, was collected via the online survey software Qualtrics (Qualtrics; Provo, UT). Control questions regarding ongoing pregnancy, previous childbirth, wish to give birth in the future, and preferred mode of birth were also included to ensure that participants met our target group criteria. Of the 42 individuals who submitted the registration form, 33 provided contact information and met the initial eligibility criteria. These criteria included: being 18 years old or older, having previously given birth, being non-pregnant at the time of recruitment, expressing a desire for one or more additional children, and hesitating to pursue another pregnancy due to FOC. Of the 33 eligible participants, 15 were purposefully selected by predefined criteria to ensure variation in age, educational level, and previous mode of birth, as these factors were considered relevant for capturing diverse experiences of FOC. Selecting 15 participants allowed for potential non-responses or withdrawals. The 15 selected participants were invited to take part in the interview study, either by phone or e-mail, and received both oral and written information about the study, with the opportunity to ask questions. When seven of the invited participants declined, we proceeded with the eight who agreed to participate because they represented the intended variation in age, educational level, and birth history. Additional interview invitations were not considered necessary, as these eight participants provided rich and diverse data sufficient to explore the research aim in depth. After the interviews were completed, all individuals who completed the registration form but were not invited to the study were notified that the recruitment process had been completed.

### Participants

Eight participants, aged 27 to 35 years, were interviewed. All were born in Sweden and were either married or cohabiting. One lived in a small town (approximately 10,000–24,999 inhabitants), five in medium-sized towns (25,000–49,999 inhabitants), and two in a major city (> 50,000 inhabitants). Their educational levels ranged from high school to university. The majority of participants, all of whom identified as women, had experienced one previous birth, with one participant having given birth twice. Four had given birth vaginally, and six had experienced FOC during their last pregnancy, with three receiving counseling for it. No clinical evaluation of traumatic birth experiences or posttraumatic stress was carried out. All participants expressed a longing for one or more additional children but felt hesitant about another pregnancy and birth due to FOC. Two women had decided not to become pregnant again because of their fear, whereas the other six women were ambivalent. Among these six women, three wished to give birth vaginally, and three preferred to give birth by cesarean section for a future pregnancy (see Table 1).

**Table 1:** Participants’ demographic information

Participant pseudonyms	Previous births		Birth mode	FOC in the previous pregnancy	FOC counseling during previous pregnancies	Previous traumatic birth experience^a^
Emma		1	Vaginal birth	Yes	No	Yes
Olivia		2	Elective cesarean sections (two upon request from the mother)	Yes	Yes (twice)	No
Sofia		1	Vaginal birth	Yes	Yes	No
Alice		1	Vaginal birth	Yes	No	Yes
Julia		1	Emergency cesarean section	No	No	Yes
Clara		1	Vaginal birth	Yes	Yes	Yes
Anna		1	Emergency cesarean section	No	No	Yes
Elsa		1	Elective cesarean section (for medical reasons)	Yes	No	No

Note. ^a^Previous traumatic birth experiences were not clinically evaluated but self-defined in reference to experiences of interactions and/or events directly related to childbirth that caused overwhelming distressing emotions and reactions, in line with the definition of Leinweber et al. 2022

### Data collection

The semi-structured interviews were conducted in Swedish by the second author in February and March 2020. An interview guide, developed by the authors, focused on participants’ experiences of FOC, childbirth, and how they coped with FOC. The guide was tested in a pilot interview with an individual who was not eligible for the study, as she anticipated being able to become pregnant and give birth again. No changes were made following the pilot interview, and the data from that interview were not included in the study.

For the participants’ convenience, they could decide on the timing and mode of the interview – either face-to-face, by video link, or by phone. Two interviews were performed via video links, and the remaining six were conducted via the phone. Before the interviews commenced, all the participants received oral information about the study and had the chance to ask further questions. Thereafter, verbal consent was obtained. The interviews, which lasted 41 to 92 min, were recorded via a Dictaphone, and then transcribed.

### Data analysis

We used reflexive thematic analysis according to Braun and Clarke [30] to analyze the data, starting from an experiential perspective and conducting the analysis inductively and at a manifest level. Following Braun and Clarke’s six-phase process, the first and second authors started by reading all the transcripts repeatedly to familiarize themselves with the data. Thereafter, the second author performed the initial manifest and inductive coding by identifying and marking pieces of text and linking them to codes, under the supervision of the last author. Next, the first author developed initial themes by searching for patterns among the codes. These initial themes were then reviewed by the first and last authors to ensure that the codes within each theme were centered around a coherent concept, distinct from codes relating to the other themes, and clearly represented in the full transcripts. The themes were then discussed, refined, defined, and labeled. After the first author had written a first draft of the results, the theme names were refined once more, and quotes were selected and translated to English to illustrate the findings for each theme.

## Results

In our analysis, we identified five themes, which we summarized under the overarching theme: “*In the grip of fear*,* striving to find a way out*.” This theme reflects the intense FOC experienced by women who were not currently pregnant. Despite their strong desire for more children, this fear led them to delay or avoid future pregnancies. The fear often originated before or during their first pregnancies and was sometimes intensified by traumatic childbirth experiences. The women reported a lack of professional support when they were not pregnant, and the absence of an individualized approach left them feeling isolated and unsupported. These experiences significantly impacted their lives and reproductive futures, illustrating their ongoing struggle to escape the grip of fear.

**Fig. 1:**
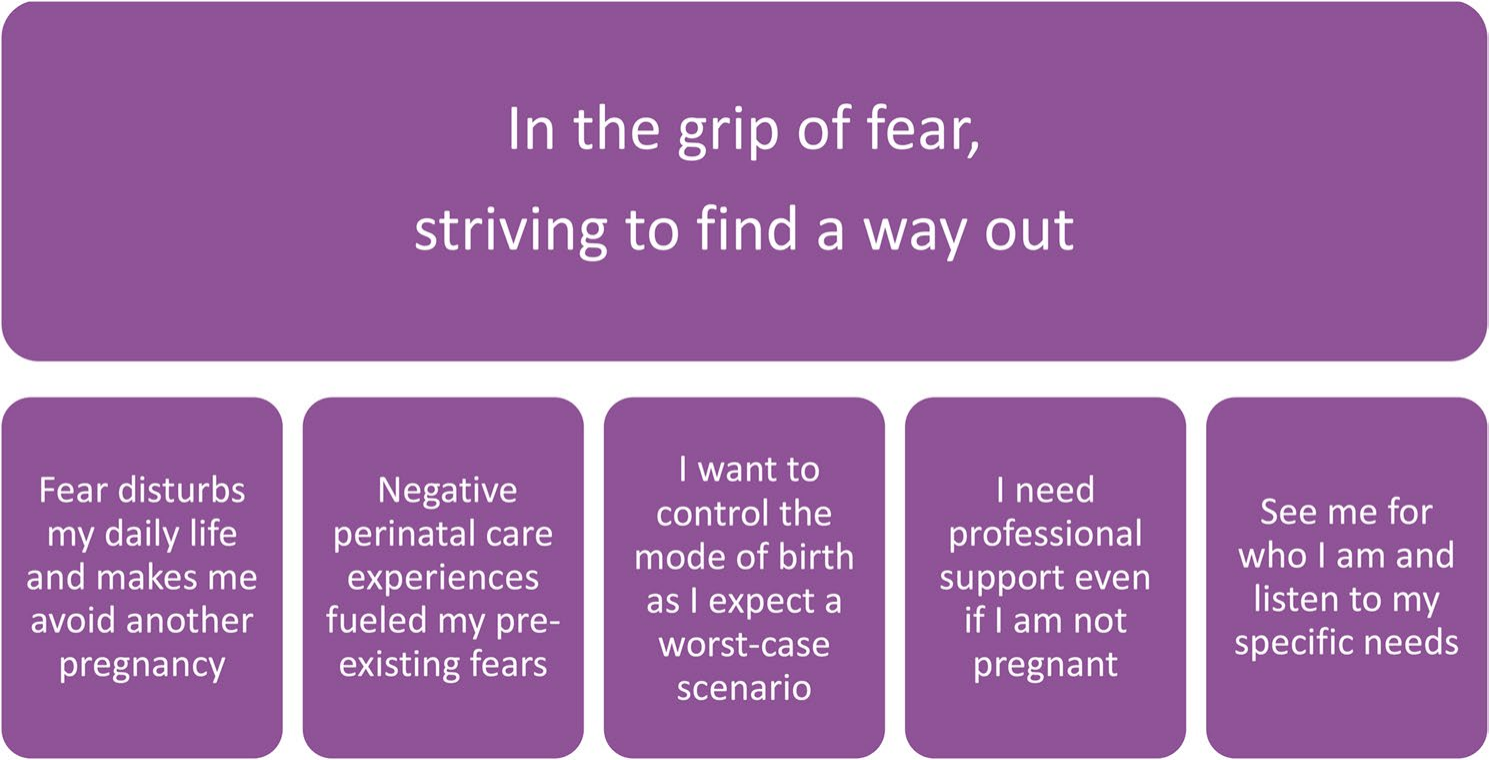
Overarching theme and themes

The overarching theme was manifested in five themes: “*Fear disturbs my daily life and makes me avoid another pregnancy*,” “*Negative perinatal care experiences fueled my preexisting fears*,” “*I want to control the mode of birth as I expect a worst-case scenario*,” “*I need professional support even if I am not pregnant*,” and “*See me for who I am and listen to my specific needs*” (see Fig. 1).

### Fear disturbs my daily life and makes me avoid another pregnancy

All women in this study described intense feelings of fear that were hard to manage; for some, the experience was more like panic or a sense of impending death. It affected the women’s daily lives in different ways. They reported feeling stressed and depressed, having difficulties sleeping and frequent nightmares, and worrying about the future – for themselves, as well as for their existing or future children. One woman described that she now became frightened when experiencing menstrual pain, as it reminded her of her femininity and the possibility of becoming pregnant again.

*I’ve never been able just to be and feel that everything is just fine; instead*,* I am constantly reminded that I am a woman and that I can get pregnant. And if I don’t get pregnant*,* I still have a longing to have children /…/ but I’ve realized that I become afraid when I get menstrual pain.* (Alice)

Above all, fear hindered the women from trying to become pregnant again, despite a strong desire to have more children in the future. While they expressed a deep gratitude for the children they already had, many also wanted to give their child a sibling. Some chose to postpone thinking about childbirth, but as time passed, their longing for more children grew stronger. The women described weighing the emotional toll of enduring nine months of psychological suffering and the risk of a traumatic birth experience against their desire to have another child.

The women had either postponed a potential pregnancy due to FOC or decided never to become pregnant because of their fear. In some cases, abortion, dual contraceptive methods, or sterilization became ways of avoiding pregnancy.

*It [the abortion] was about a year and a half after my first child. At that moment*,* I felt ‘no*,* I can’t handle this now’ /…/ I still had too much to process within myself.* (Anna)

When pregnancies were postponed, the women felt that their window of opportunity to have more children was beginning to close, and they worried that one day it might be too late. Some women said that they would have liked to have one or two years between the children, but now many years had passed since the first child was born. Women who had experienced a miscarriage described mixed emotions, such as relief alongside sadness.

*At the same time as I was sad because there was no baby*,* I was also relieved*,* thinking*,* ‘oh shit*,* I don’t have to give birth. I don’t have to experience that again’. (*Alice)

While struggling with the decision, the women felt an overwhelming expectation from others to have more children, even though they knew that their last birth had been difficult. Having one child often made others ask, “when is it time for the next child?”

Some of the women had support from family and friends and could talk about their fears and ambivalence openly. Sharing their experiences brought a sense of relief and made their situation easier to manage. Many thought it was important to talk about these issues and did so actively. However, although they generally felt comfortable discussing the issue, they avoided discussing it with certain individuals, particularly those without children or those who were currently pregnant.

In general, women felt supported by their partners and talked to them about their fears. Nonetheless, some women described their partners as also being afraid of childbirth, making it difficult for them as a couple to address the issue together. Consequently, women also worried about their partners, who had to cope with their own fear, as well as the women’s fear and ambivalence regarding having more children. This made the women’s struggle even harder.

To manage their difficult situation, the women described using several strategies to address their fears and take care of themselves. Some found that pursuing hobbies helped reduce their fear, as it served as a breathing space that facilitated emotional processing. Several women also sought ways to gain insight and learn more about childbirth. One approach was to process a previous childbirth experience by reading about the complications that arose during childbirth or seeking medical care to obtain an explanation for why the birth turned out as it did. Many women managed their fear by searching for information through various networks. Some read about others’ experiences on social media or connected with women in similar situations via online forums; in contrast, others borrowed academic literature about childbirth or read scientific reports. A few women perceived the tone of some self-help books on FOC as patronizing, which led them to prefer factual resources instead. Additionally, some women tried to manage their fear by listening to podcasts about childbirth.

Gathering information and engaging with other people’s birth stories was a strategy that had both advantages and disadvantages. Reading about negative birth experiences and media reports about understaffed maternity wards and a shortage of hospital beds often increased anxiety. However, social media and podcasts also provided strength, comfort, support, and up-to-date information regarding pregnancy and childbirth.

*Then I can almost feel as if I gain strength from reading and hearing about others and understanding that I am not alone in my situation*,* that there are others who share the experience /…/ And that has made me feel that there is nothing wrong with me.* (Julia)

### Negative perinatal care experiences fueled my preexisting fears

Most women described that their fear had been present even before their first pregnancy, leading some to deliberately wait before having their first child. When they later decided to become pregnant, increasing age and concerns about reproductive longevity were important factors. For others, fear emerged or worsened after giving birth, when they fully understood what they had experienced during labor. Feeling that childbirth was more difficult than anticipated, or having experienced mistreatment from healthcare professionals (HCPs) during birth, were examples of factors that helped fuel the fear.

Those who had wanted to give birth by cesarean section in a previous pregnancy described a difficult struggle to secure a planned cesarean birth. Several women reported that HCPs promoted vaginal birth and tried to convince them to opt for it, even though they desired a cesarean section. One woman had read scientific articles about cesarean sections to use as ammunition in her fight to be granted a cesarean section, which gave her the confidence to argue her case.

These women also experienced stigma and preconceived notions from the people around them. They felt an expectation that births should be vaginal, with cesarean births often seen as a loss for the mother or an “easy way out.” Waiting to receive a response to their request for a cesarean birth was described as damaging the women’s quality of life. The women explained how they could not stop thinking about the birth or relax until their request was granted. Receiving a decision about a planned cesarean birth felt like great relief, even though it often came (too) late in pregnancy.

*So*,* when people ask*,* ‘Well*,* how long did it take you to give birth?’ I usually say it took eight months*,* because it took eight months of struggle*,* in some way*,* to get… to fight for the birth I wanted.* (Olivia)

Most women had negative experiences during their last pregnancy or birth, which resulted in feelings of not wanting to go through the same situation again. These negative experiences occurred during pregnancy, early labor, the active phase of labor, or the postpartum period. They were characterized by feelings of panic, fear, anxiety, pain, hopelessness, loss of control, a sense of having a body incapable of giving birth, and a lack of understanding about what was happening. Several women had experienced prolonged labor, which intensified those feelings even more.

Several women felt that HCPs normalized their difficult feelings and/or the distressing events that occurred during pregnancy, birth, and the postpartum period. As a result, the women felt they were not taken seriously. They described experiences of not feeling understood during their encounters with HCPs, and expressed that HCPs did not believe they knew their own bodies or what was best for them. This resulted in feelings of being diminished and not receiving the help they felt they needed, which, in turn, caused a loss of trust in midwives and a general distrust in the healthcare system.

*Mentally*,* I felt very unwell after this birth /…/ It was also like*,* ‘well*,* everyone feels bad afterward’ /…/ so no one took it seriously either.* (Emma)

Others had experienced shortcomings in treatment during pregnancy, birth, or follow-up care. Not feeling safe during birth was a significant issue, stemming from not being listened to, being ignored, and perceiving HCPs as unprofessional. After giving birth, some women felt they had received both inadequate and insufficient support and treatment. Women felt that they needed immediate and regular support to recover after a traumatic birth experience, which they did not receive. This resulted in difficulties bonding with their newborns and complications with breastfeeding.

*I also felt like*,* well*,* now this baby is here and getting all the attention. I’m here too*,* I want support as well /…/ She screams and cries when she needs help*,* but should I lie on the floor and scream and cry to get help too. Or what should I do to get help?* (Olivia)

Several women also stated that the lack of resources and competence they encountered in maternity care contributed to their current fears. The lack of competencies they mentioned were both medical and psychological, skills they felt all HCPs working with birthing women should possess. The women believed that if they had been treated differently during and after their last pregnancy and birth, their current situation would probably have been significantly better. They provided several suggestions for how HCPs could have acted differently. Improved information-sharing between caregivers and healthcare facilities was emphasized, particularly to ensure that all involved professionals were informed about the woman’s situation, so that the woman did not have to repeatedly explain it. In addition, they requested more attentive and empathetic communication from HCPs, professionals who truly listened and tried to understand.

*They could have listened better to what I was actually saying*,* like trying to understand more – what is she trying to convey? There were quite a few people around me*,* but in my mind*,* there was no one. In my mind*,* I was completely alone.* (Alice)

In contrast to the negative experiences, some women had consistently positive experiences with care and great confidence in their HCPs, particularly midwives. They felt that the HCPs genuinely cared about them, supported them, took their fears seriously, and tried to help them manage those fears. Women specifically mentioned that they appreciated HCPs who had read their medical records beforehand, showed interest in their personal stories, were fully present both physically and mentally, listened actively, offered specific suggestions for how to manage their fears, and came to visit them or called them postnatally. Given these experiences, whether positive or negative, these women valued being taken seriously, being treated respectfully and professionally by understanding HCPs. These factors contributed to their sense of safety and trust during childbirth.

### I want to control the mode of birth as I expect a worst-case scenario

When thinking about giving birth again in the future, the women expected the worst to happen. Some of the worst-case scenarios they mentioned included feeling abused, not being listened to, being unable to cooperate with the HCPs, feeling the need to emotionally shut down, giving birth prematurely, not wanting to hold the baby, the baby getting injured or dying during birth, or even seeing oneself dying during childbirth.

Women had different thoughts about how they would like to give birth in the event of a future pregnancy. Some women were ambivalent and did not know which mode of birth they would prefer. Others wanted to give birth vaginally and emphasized that there are several benefits to giving birth vaginally. For example, they hoped that a second birth would be less frightening since they knew what to expect, and they imagined having a positive birth experience the next time. Some found comfort in the belief that the second birth is usually experienced as easier than the first one.

*It is often said that the second birth tends to be easier and faster/…/ So that’s what I’m hoping for*,* but at the same time*,* when I think back on everything I’ve been through*,* I feel like “never again.”* (Clara).

Other women could not imagine giving birth in any way other than by cesarean section and felt that it was the healthiest choice for themselves and their families. They perceived cesarean births as predictable and safe because they take place under controlled conditions with competent HCPs who are constantly present and could handle any complications that may arise, unlike vaginal births, where there are large variations in the process. As a result of their previous experiences, the women who wanted a cesarean section were well aware of the difficulties in getting it granted. Even if their FOC was documented in their medical records, a planned cesarean section was rarely granted until late in pregnancy, which worsened the women’s agony. They would have to live with their fears for several months during pregnancy, and once the decision was made, they realized that it would probably be too late to have an abortion. The women wished there could be a way to decide on a planned cesarean section before even becoming pregnant, or at least in early pregnancy.

*I have to learn how to deal with living in uncertainty in some way because I know I won’t agree to a vaginal birth*,* and I just need to come to terms with learning to wait for the decision /…/ At first*,* I wanted a decision before [the pregnancy]*,* but that’s not possible.* (Elsa)

### I need professional support even if I am not pregnant

In the present situation, when not pregnant, it was difficult for women to know where to seek help. In order to dare to become pregnant, women sought help from different HCPs, such as primary care or FOC units. Some women had been referred to a psychologist, whereas others had been rejected without further guidance.

*I’m so afraid now that I don’t even want to get pregnant. I contacted the FOC unit*,* but they basically said*,* “Yeah*,* get in touch with us when you’re pregnant”/…/ I asked if I could see the psychologist at the maternity clinic*,* but they were like*,* “No*,* you can come when you’re pregnant.”* (Elsa).

Some women had avoided or postponed seeking help for their fears. They emphasized how difficult it can be to reach out to HCPs when feeling unwell and suggested that more routine follow-ups after childbirth could be beneficial. Some still felt the need for support in coping with and understanding their fear, despite the time that had passed since their last birth.

Even when pregnant, the women knew it could take some time to receive support. Although the first visit to the midwife occurs early in pregnancy, it felt too far in the future when fearing childbirth, and the following visits felt too spaced out. They specifically asked for more frequent visits to the antenatal care midwife in early pregnancy and for the midwife to ask about FOC and mental health more often.

*You have very infrequent visits at the beginning at the maternity clinic/…/ It might be important to reach out to these women right away.* (Sofia)

After giving birth, women felt that FOC and mental health problems, as well as medical complications, should be taken more seriously. They requested additional follow-up care, both during the hospital stay and afterward. Women expressed that a follow-up with the midwife who assisted with the birth should be a standard procedure. They also suggested that additional follow-ups and screening for FOC, depression, and symptoms of posttraumatic stress should be encouraged, not only two months postpartum but also later on.

*It’s been nine months since I had my baby*,* but it’s only now*,* in the last month*,* that I’ve realized I might need to talk to someone about it/…/and it’s up to me to take the initiative*,* but I don’t really know where to turn.* (Clara)

### See me for who I am and listen to my specific needs

The importance of respecting the individual was frequently stressed during the interviews. Women emphasized that HCPs being present and understanding, and treating each person as an individual could have made a significant difference in how they felt now. Some women described that HCPs withheld information about complications related to vaginal birth and focused primarily on the risks associated with cesarean births. They wished for more explicit and honest information.

*If you explain more about it*,* then you’re more prepared/…/ If you choose to go through a vaginal birth*,* then there’s nothing to be afraid of because you’re prepared for it too/…/ But right now*,* it feels like they really want to refine it [the information] and they don’t mention the possibility of tearing - they don’t say anything about it beforehand*,* and how it impacts your life afterward.* (Emma)

During previous pregnancies, some women had felt empowered by having their wishes documented in their medical records, while others valued the opportunity to visit the labor ward. Women also highlighted the benefits of attending a support group during pregnancy, designed for individuals experiencing FOC. Those who had the opportunity to see a midwife or psychologist at a FOC unit during or after pregnancy felt that it helped them manage their fear. They particularly appreciated when the HCPs were direct in their communication, gave honest and clear answers, and set specific goals for the counseling. Reviewing their previous birth with a midwife or doctor was also considered helpful.

*I see a psychologist who is helping me. She asks questions /…/ She listens to what I say*,* but if she doesn’t understand*,* she keeps asking. We also made a plan*,* like “why are you here?”*(Alice).

Not all the interventions offered by HCPs were perceived as helpful. Several women felt that the help they received did not match their wishes and needs. The examples mentioned included not receiving answers to their questions or not having enough time to address important matters. One of the women described doing everything that HCPs wanted her to do in addressing her fear, feeling that she turned herself inside out for the HCPs without seeing any results. Another woman had received counseling from a FOC unit to reduce her fear but found it unhelpful, as it felt standardized and superficial. She wanted personalized support based on her unique circumstances.

*I didn’t feel like those conversations were meant for me; they felt very standardized. They had one response that they could give to women who were afraid*,* and then they repeated the same response over and over again. They could never go in-depth*,* and all those conversations were somewhat superficial. And I got a bit provoked by that*,* so I tried to question those responses*,* and in that moment*,* I was very angry.* (Julia)

Maternity care was also considered inflexible, with only one path and no exceptions made. Women asked for alternatives, not only regarding the mode of birth but also alternatives to the whole maternity care structure. For example, they asked for caseload midwifery with continuity of care. The importance of having adequate resources in maternity care was also emphasized.

*I think fewer women would have been afraid if they had had the opportunity to have more influence*,* and not just I’m a strong advocate for cesarean sections*,* but also to have a say in how a vaginal birth should proceed. And that’s what’s difficult; you can’t influence a healthcare system that is barely functioning.* (Elsa)

## Discussion

This study explored the experiences of women with FOC who had given birth previously but were not currently pregnant. Although these women expressed a desire for more children, their fear led them to either postpone future pregnancies or decide against becoming pregnant again.

The findings highlight the internal conflicts these women face as they are confronted with the decision to potentially become pregnant again or to avoid future pregnancies altogether. Within this struggle, they describe several key experiences related to FOC. First, most women reported experiencing FOC during their first pregnancies as well, although it was not always acknowledged at the time. Many described their childbirth experiences as traumatic, which contributed to a deepening of their fears over time. Throughout both pregnancy and postpartum, they felt that the care they received lacked an individualized approach and instead followed a rigid, by-the-book model. Furthermore, the women felt overlooked, as they were neither asked about their fears nor offered help when their fears were acknowledged. The lack of available resources led to feelings of isolation. These experiences had a profound impact on their current lives and contributed to their reluctance to pursue future pregnancies.

Our findings indicate that it would be an oversimplification to attribute parous women’s FOC solely to previous traumatic birth experience. In many cases, FOC had also been a part of their experience in a previous pregnancy. This suggests that, although these women might typically be classified as having secondary FOC, many may also have demonstrated signs of primary FOC. The question of whether the distinction between primary and secondary FOC is the most useful has been debated [31], and our findings further support the notion that this classification may not always be the most beneficial.

A traumatic birth experience often leads to FOC [32] and is also a well-documented outcome of FOC [33, 34]. Interestingly, the women’s experiences in this study resemble the fears described by non-pregnant women with FOC who have never given birth. These include feelings of panic, a total loss of control, and a sense of being physically or mentally incapable of giving birth [4]. This similarity suggests that many of the fears associated with FOC may remain consistent, regardless of actual childbirth experience.

The importance of respecting the individual was frequently expressed during the interviews. Women shared that having HCPs who were present, understanding, and treated them as individuals could have significantly impacted their current feelings. Their desire for a more individualized approach also included care routines during both pregnancy and childbirth. However, the available options were perceived as limited, with the only alternative to a standardized vaginal birth being a cesarean section. It seems reasonable that these women, similar to those in a study from the United Kingdom [UK], try to avoid previous negative experiences and seek options that allow them to maintain a sense of control [35]. In the UK, women have the right to make their own decisions regarding how and where to give birth [36]. This autonomy is reflected in their varying preferences expressed for future births, including vaginal hospital birth, home birth, planned cesarean section, or even freebirth. Thus, expanding the range of available birth options and fostering an environment that prioritizes individualized care and respects women’s autonomy may enhance childbirth experiences and overall satisfaction, particularly for women fearing birth.

Building on this, our findings reveal that when women sought to exercise such autonomy through requesting a cesarean section, they often encountered barriers. They described long negotiations and decisions late in pregnancy, despite documented FOC. This struggle negatively affected their quality of life and reinforced feelings of stigma, as vaginal birth was perceived as the expected norm. Similar patterns have been described previously in Sweden, where a strong belief in normal birth and skepticism toward FOC as an indication for elective cesarean section prevail [28]. Recent research emphasizes the need for structured and ethically grounded approaches to maternal requests for cesarean sections in Sweden, including unbiased information and exploration of underlying reasons beyond FOC, such as bodily autonomy [37]. At the same time, cesarean section on maternal request entails increased healthcare costs, which likely contributes to the restrictive policies [38]. Together, these findings highlight the need for maternity care that offers a wider range of options and consistently respects women’s preferences, regardless of whether these involve homebirth, caseload midwifery, planned cesarean section, or other individualized approaches. Achieving this requires transparent communication, shared decision-making, and national policies that enable informed choices within the framework of safe and equitable care.

Our participants also encountered several barriers in obtaining professional support; some were unaware of where to seek care, whereas others avoided it because of the emotional burden, or simply did not receive support because they were not currently pregnant. While antenatal education and FOC support is routinely offered to pregnant women in Sweden, access to support is highly limited when not currently pregnant. According to a meta-synthesis focusing on pregnant women with FOC [39], receiving support for FOC can empower women, reduce their fears, and have a healing effect. For this support to be beneficial, women must feel heard, validated, and respected. It was also found that focusing on the future after giving birth can be beneficial [39], a strategy that could also be valuable for women who are not pregnant.

Consistent with the conclusions drawn by Olsen et al. [34], who studied pregnant women with FOC following a traumatic birth, our findings strengthen the understanding that traumatic birth experiences often need to be actively processed to prevent the development of FOC and further traumatic experiences when giving birth again. A recent study mapping policies and services for women following a traumatic birth identified only one country (the Netherlands) that had national policies regarding screening, treatment, and prevention of traumatic births [40]. This highlights a significant gap in national policies and postpartum follow-up for women who have traumatic birth experiences.

An American study emphasized that, due to fragmented postpartum care, all professionals who interact with a woman after childbirth must be attentive to potential signs of birth trauma [41]. This aligns with our findings, in which women expressed a strong need for more comprehensive follow-up postpartum, not only around two months after childbirth but also at later stages. Addressing these gaps in care and policy is crucial for preventing FOC and supporting women’s mental health after a traumatic birth experience.

### Strengths and limitations

This qualitative study successfully met its aim; however, the number of participants was relatively small. Although a larger and more diverse group of participants would have been desirable, the participants in this study differed in educational level, birth experiences, and future intentions, including whether they wished to have more children and their preferred mode of a potential future birth. Some had experienced FOC before or during previous pregnancies, whereas others had not. A more diverse group may have provided an even broader range of insights. Conducting interviews over phone or video calls gave the opportunity to recruit across a larger geographical region and including participants giving birth and receiving support in different parts of the country. Although video interviews may have been beneficial to build rapport and increase engagement in the conversation, we followed participant preferences, which most often were to interview over the phone. Data were collected just before the COVID-19 pandemic drastically impacted perinatal care in Sweden. Hence, any changes in the experience of FOC that may have occurred during or after the pandemic are not captured in our data. On the other hand, the experiences described were not influenced by the extreme birthing circumstances that characterized the pandemic period.

## Conclusion

These findings highlight the urgent need for support systems that effectively address the psychological needs of women with FOC and traumatic birth experiences, both during pregnancy and in the postpartum period. Support should not be limited to the first few months postpartum or postponed until a subsequent pregnancy. Every woman who has given birth should have access to evidence-based support, regardless of how much time has elapsed since the birth. To help women manage their FOC and rebuild confidence in their ability to give birth, it is essential to respect their individual choices and preferences. Maternal care providers must be equipped to offer a broader range of options within maternity care to meet these needs.

## Supplementary Information

Supplementary Material 1.

## Data Availability

The datasets generated during the current study are not publicly available due to the risk of compromising individual privacy. However, excerpts of the interviews can be made available by the authors upon reasonable request.

## Abbreviations

FOC: Fear of childbirth
HCPs: Healthcare professionals
