# Supplementary material for: The lasting effects of fear of childbirth: parous women’s experiences of postponing or avoiding subsequent pregnancies
Source: Reprod Health. 2026 Jan 9;23:22. doi: 10.1186/s12978-025-02261-1 (PMC12849174; doi:10.1186/s12978-025-02261-1)
Supplement: Supplementary file 1 — Supplementary Material 1. [file 12978_2025_2261_MOESM1_ESM.docx]

The lasting effects of fear of childbirth: Parous women’s experiences of postponing or avoiding subsequent pregnancies, Ternström et al. 2026.

Appendix 1: Interview guide

**Fear of Childbirth**

How would you describe your fear of childbirth?

What do you think has contributed to your fear?

How does this fear affect your daily life?

Is there anything you do to try to reduce your fear?

If you imagine that you would give birth, what do you see in your mind?

**Previous Childbirth Experiences**

Can you describe your previous experiences of pregnancy and childbirth?

How was your experience of maternity care?

Has your fear been influenced by your previous experiences? If so, how?

**Expectations or Perceptions of Childbirth**

What thoughts and feelings arise wanting more children but not daring?

Do you feel that you can talk to others about not daring to have more children?

**Treatment and Support**

Have you sought help for your fear?

Is there anything the healthcare system could have done differently for you, based on your previous birth experiences?

Is there anything you would need in order to feel comfortable seeking care?

Are there any barriers or difficulties that prevent you from seeking care?

What kind of support would you wish to receive from healthcare providers?
